# Epstein-barr virus-associated hemophagocytic lymphohistiocytosis after antithymocyte globulin-conditioned allogeneic hematopoietic stem cell transplantation: a single-center case series

**DOI:** 10.1007/s00277-026-07307-3

**Published:** 2026-10-17

**Authors:** Ingerid Weum Abrahamsen, Anders Eivind Myhre, Camilla Dao Vo, Tobias Gedde-Dahl, Tor Henrik Tvedt

**Affiliations:** 1 Department of Hematology, Oslo University Hospital, Sognsvannsveien 20, Oslo, 0372 Norway; 2 Institute of Clinical Medicine, University of Oslo, Klaus Torgårds vei 3, Oslo, 0372 Norway

Hemophagocytic lymphohistiocytosis (HLH) is a severe hyperinflammatory syndrome associated with multiorgan failure and a high mortality rate [1]. In adults, HLH is commonly triggered by acquired conditions such as malignancies, infections, autoimmune diseases and therapies that induce immune dysregulation. A substantial proportion of cases are associated with immune dysregulation in combination with reactivation of the Epstein–Barr virus (EBV) [2]. 

In allogeneic hematopoietic stem cell transplantation (allo-HSCT), EBV reactivation is a well-recognized complication. EBV-reactivation may lead to post-transplantation lymphoproliferative disease (PTLD), which is also a potentially life-threatening complication [3]. EBV-driven HLH is rare and associated with very high mortality [4–7]. Because existing evidence is limited to case reports and small case series, optimal diagnostic and treatment strategies remain poorly defined. 

We retrospectively identified cases of EBV-triggered HLH (EBV-HLH) in allo-HSCT recipients at our institution. HLH is not registered in our allo-HSCT registry. Case identification employed extracting patients with EBV reactivation and plasma ferritin above 20 000 µg/L from the registry as well as manual patient identification. Diagnosis was confirmed by review of electronic patient records, with HLH defined according to the assessment of the treating physician. The last data extraction was done March 2026. The local allo-HSCT registry is a consent-based database. The registry has been reviewed and approved by the institutional Data Protection Officer and the publication of the registry data has been approved by the Regional Ethics Committee (approval no. 2019/614).

Using an arbitrary cutoff of plasma ferritin > 20 000 µg/L 51 patients were extracted from the transplant registry. Of these 7 were EBV-HLH cases. Another 4 probable cases were identified but could not be included due to missing diagnostic data. Of the remaining cases, 23 were sepsis, 9 liver graft-versus-host disease (GvHD) or veno-occlusive disease, 5 other inflammatory conditions and 3 HLH not related to EBV. Another 3 EBV-HLH cases were manually identified, 2 as HLH was treated at the referring hospital resulting in incomplete laboratory data in our registry, 1 because plasma ferritin was below 20 000 µg/L. As the main purpose of the study was to describe clinical cases, and not to estimate the incidence of HLH, a lower threshold for plasma ferritin > 20 000 µg/L was not tested. Thus, a total of 10 cases are described. 

The data extraction included 1470 patients undergoing 1549 transplants between August 2010 and March 2026; 930 transplants (903 patients) incorporated antithymocyte globulin (ATG) as GvHD-prophylaxis. Ten EBV-HLH patients were identified, yielding an incidence of 1.1% among the ATG exposed patients.

H-score could be retrospectively calculated in 8 of 10 patients, although 3 of the 8 lacked bone marrow sampling and received score 0 for this variable [8]. All 8 patients had an H-score above 200, corresponding to a probability of HLH greater than 90%. The 2 remaining patients were treated for HLH at referring hospitals, and all laboratory values were not available.

All 10 patients were transplanted for malignant diseases: acute myeloid leukemia (7 patients), acute lymphoblastic leukemia (2 patients) and myelodysplastic syndrome (1 patient). Median age was 55 years (range 16-70), 5 males and 5 females. All 10 patients were transplanted using unrelated donors, both HLA-matched (MUD) (6 patients) and mismatched (MMUD) (4 patients). A total of 9 patients received peripheral blood stem cells, 1 received bone marrow. All 10 patients received ATG as part of their GvHD prophylaxis, 9 received Thymoglobulin (2 mg/kg/day, days -2 to –1 for MUD and –3 to –1 for MMUD), whereas 1 patient received Grafalon (15 mg/kg/day days -3 to -1). None of the patients had GvHD, 1 had cytomegalovirus reactivation. All 10 patients were EBV seropositive and were transplanted with EBV seropositive donors.

EBV reactivation occurred at a median of 52 days post-transplant (range 27-67). The median EBV-DNA load in whole blood was >1 000 000 IU/mL (range 875 000->1 000 000, values above the upper limit of quantification were right-censored at the assay ceiling of 1 000 000 IU/mL). The diagnosis of HLH was in most cases delayed by days or weeks but retrospectively occurred shortly after EBV-reactivation. Median plasma ferritin was 107,500 µg/L (range 13 000-326 000). All patients were diagnosed with PTLD with an adequate histological sample, 1 post-mortem. One patient had polymorphic PTLD, 1 had indeterminate, the remaining were monomorphic PTLD. 

EBV-HLH was treated with rituximab and corticosteroids in all 10 patients. A total of 6 patients received chemotherapy, 6 patients received intravenous immunoglobulins and 5 patients received anakinra. A total of 4 patients were treated with tocilizumab and 2 with ruxolitinib (Fig. 1).

**Fig. 1:**
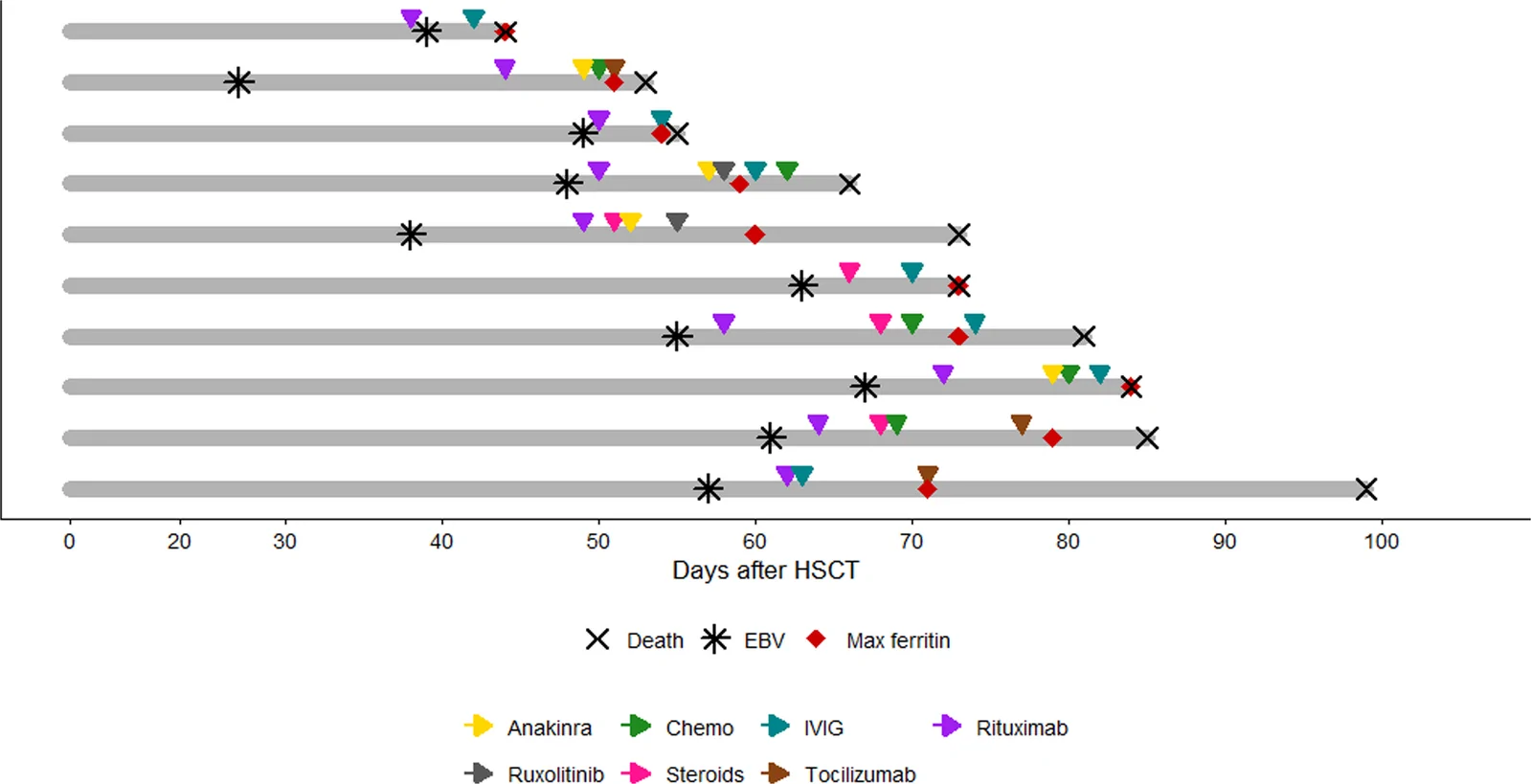
Swimmer plot of each patient’s clinical course including treatment. Each horizontal bar represents one patient. Bar length corresponds to time from transplant (day 0) to time of death (depicted as X). Symbols indicate timing of key clinical events: EBV reactivation (diagnostic sample), maximum s-ferritin level, first dose of the different treatments and death. HSCT: hematopoietic stem cell transplantation, IVIG: intravenous immunoglobulin, Chemo: chemotherapy

All 10 patients died, with a median time to death of 70 days (range 44-99). The clinical courses were uniformly dramatic with progressive multiorgan failure despite aggressive treatment and admission to the intensive care unit. The immediate cause of death was intracerebral bleeding in three patients and multiorgan failure in the remaining patients.

Our 10 clinical cases show striking similarities. EBV-HLH occurred within three months post-transplant, exclusively in patients treated with ATG and with EBV-seropositive donors, and all 10 patients had clinical and histological verified PTLD. Despite aggressive treatment, all died within a few weeks following the diagnosis of EBV-reactivation. 

Our data does not permit estimation of the true incidence of EBV-HLH following allo-HSCT. Underdiagnosis is very likely, as the cardinal features of HLH, fever and systemic inflammation, overlap with EBV/PTLD itself and with common transplant complications such as GvHD and infections [7]. Additionally, less severe cases may resolve without an HLH diagnosis, when treatment of the EBV/PTLD trigger proves sufficient.

Treatment of secondary HLH requires both treating the underlying trigger, as well as dampening the hyperinflammatory responses [1]. When EBV-PTLD is diagnosed in the setting of allo-HSCT, patients receive rituximab alongside reduction of immunosuppression, the latter being considered important, given that adequate T-cell immunity is generally required for long-term EBV and PTLD control [3]. As discussed by Ali et al [4] this creates a therapeutic dilemma when HLH coexists with EBV-PTLD. Reduction of immunosuppression is necessary to allow T-cell recovery, but risks exacerbating the hyperinflammatory state. EBV-specific T cell therapy could potentially resolve this issue but is not readily available at our center. Both PTLD and HLH are often clinically aggressive conditions, allowing little time for balancing treatments to obtain both immune reconstitution and control of hyperinflammation. Evidently, the result is a very high mortality rate. 

Early diagnosis and intervention of HLH in the context of EBV-reactivation is likely critical for patient outcomes. However, dedicated scoring tools and validated biomarkers for secondary HLH in the immunocompromised host are lacking, and the widely used H-score was derived from and validated in immunocompetent patients [8]. Specific diagnostic tools for this population are warranted. We suggest that plasma ferritin should be included in the work-up at the time of EBV-reactivation, and that there should be a low threshold for full HLH work-up in patients with markedly elevated ferritin or other features suggestive of hyperinflammation. 

Preventing EBV-HLH is likely the most effective measure. EBV-reactivation is rare in non-T-cell depleted allo-HSCT [9]. The most obvious measure might therefore be to employ post-transplantation cyclophosphamide (PTCy) as GvHD-prophylaxis instead of ATG. Another preventive strategy is to administer a single prophylactical dose of rituximab during the peritransplant period. Clinical data to support this strategy are scarce, but in a recent retrospective observational study by Zhang et al 375 mg/m2 rituximab between day -15 and +30 significantly reduced the incidence of EBV reactivation, PTLD, and infection-related mortality compared to a preemptive strategy. Long-term survival was, however, similar between the two groups [10]. 

In summary, early-onset EBV-driven HLH following allo-HSCT with ATG serotherapy carries an exceptionally high mortality rate, highlighting the need for rapid diagnostic and preventive strategies.

## Data Availability

The datasets generated and analyzed during the current study are available from the corresponding author on reasonable request.
